# Colon Adenocarcinoma in the Cervical Region After Esophagocoloplasty: A Systematic Review and Case Report

**DOI:** 10.3390/medsci14060636

**Published:** 2026-10-02

**Authors:** Georgiana Gagiu, Horia-Dan Lișcu, Iman Mologani, Lucian-Ionuț Antone-Iordache, Iulia Toma, Bianca Vișoiu, Flavia Ultimescu, Gabriel Cristian Popescu

**Affiliations:** 1 Faculty of Medicine, University of Medicine and Pharmacy “Carol Davila”, 050474 Bucharest, Romania; 2 Radiotherapy Department, Colțea Clinical Hospital, 030167 Bucharest, Romania; 3 Radiotherapy Department, Emergency University Hospital Bucharest, 050098 Bucharest, Romania; 4 Oncology Department, Coltea Clinical Hospital, 030167 Bucharest, Romania; 5 Department of Pathology, Institute of Oncology “Prof. Dr. Alexandru Trestioreanu”, 022328 Bucharest, Romania; 6 General Surgery Department, Bagdasar Arseni Clinical Emergency Hospital, 041915 Bucharest, Romania

**Keywords:** colon interposition, esophagocoloplasty, adenocarcinoma, colonic graft, radiotherapy, metastatic colorectal cancer

## Abstract

**Background/Objectives**: Colon interposition, or esophagocoloplasty, remains an important reconstructive option when the native esophagus cannot be preserved or when the stomach is unsuitable for reconstruction. Malignant transformation within the interposed colon is rare but clinically relevant, because the transposed colonic mucosa retains the potential to develop adenomatous lesions and adenocarcinoma in the long term. **Methods**: We reviewed reported cases of malignancies arising in a colonic interposition after esophagocoloplasty. PubMed and Web of Science Core Collection were searched using the query “(Esophag*) AND ((Esophagectomy) OR (Esophagocoloplasty) OR (Interpos*) OR (Transpos*)) AND (Colon) AND (Cancer OR Adenocarcinoma) NOT (“Esophageal cancer” OR “Esophageal adenocarcinoma” OR “Gastric cancer” OR “Gastric Adenocarcinoma”)”. The search was limited to publications from 2005 to 2026. **Results**: After duplicate removal and eligibility screening, 20 case reports were included. We also present a 67-year-old man who underwent isoperistaltic coloesophagoplasty in 1979 after caustic ingestion during a suicide attempt and developed metastatic colon adenocarcinoma in the interposed graft approximately 44 years later. The disease was initially metastatic to the liver and subsequently progressed locoregionally in the cervical graft segment. Management included FOLFOX plus bevacizumab, de-escalation to FUFOL plus bevacizumab, FOLFIRI plus bevacizumab, cervical intensity-modulated radiotherapy with adaptive replanning, and later regorafenib. **Conclusions**: This case highlights the diagnostic and therapeutic complexity of adenocarcinoma arising in a cervical colonic graft and supports long-term vigilance, multidisciplinary evaluation, and individualized management in patients with prior esophagocoloplasty.

## 1. Introduction

Colon interposition, also known as esophagocoloplasty, is a reconstructive surgical procedure used to restore digestive tract continuity when the native esophagus cannot be preserved or when the stomach is not suitable as a substitute. Although gastric pull-up is preferred in many centers, colon interposition remains an important alternative in selected patients, particularly after caustic esophageal injury, complex benign strictures, failed previous reconstruction, prior gastric surgery, or extensive tumors involving the proximal esophagus or hypopharyngeal region [1].

The procedure is technically demanding and may be associated with early and late complications. Malignant transformation of the interposed colon is a rare but clinically significant late event. Because the transposed segment contains colonic mucosa, it retains the biological potential to develop adenomatous lesions and adenocarcinoma. Proposed mechanisms include chronic inflammation, altered luminal exposure, food stasis, reflux, mucosal irritation, and age-related carcinogenic processes, although the exact pathogenesis remains incompletely understood [2,3,4,5]. A systematic review of metachronous adenoma and adenocarcinoma after colon interposition reported a low estimated interval cancer rate, between 0% and 0.22%, while emphasizing the limited quality of cohort data and the need for lifelong endoscopic surveillance [6].

## 2. Materials and Methods

We conducted a systematic literature review of case reports of malignancies diagnosed in a colonic interposition after esophagocoloplasty. A search of PubMed and Web of Science Core Collection was performed using the query “(Esophag*) AND ((Esophagectomy) OR (Esophagocoloplasty) OR (Interpos*) OR (Transpos*)) AND (Colon) AND (Cancer OR Adenocarcinoma) NOT (“Esophageal cancer” OR “Esophageal adenocarcinoma” OR “Gastric cancer” OR “Gastric Adenocarcinoma”)”. The exclusion terms were used to improve search specificity by reducing records focused primarily on native esophageal or gastric malignancies, as the review was specifically intended to identify neoplasms arising within the interposed colonic segment.

The search was restricted to publications from 1 January 2005 to 30 June 2026, with the year 2005 selected to provide an approximately 20-year review period focused on relatively contemporary literature. Given the highly specific nature of the topic, this period yielded a substantial number of records for screening and was considered sufficient for the intended contemporary review. The final literature search was performed on 30 June 2026. No additional reference-list screening or forward citation tracking was performed.

**Figure 1:**
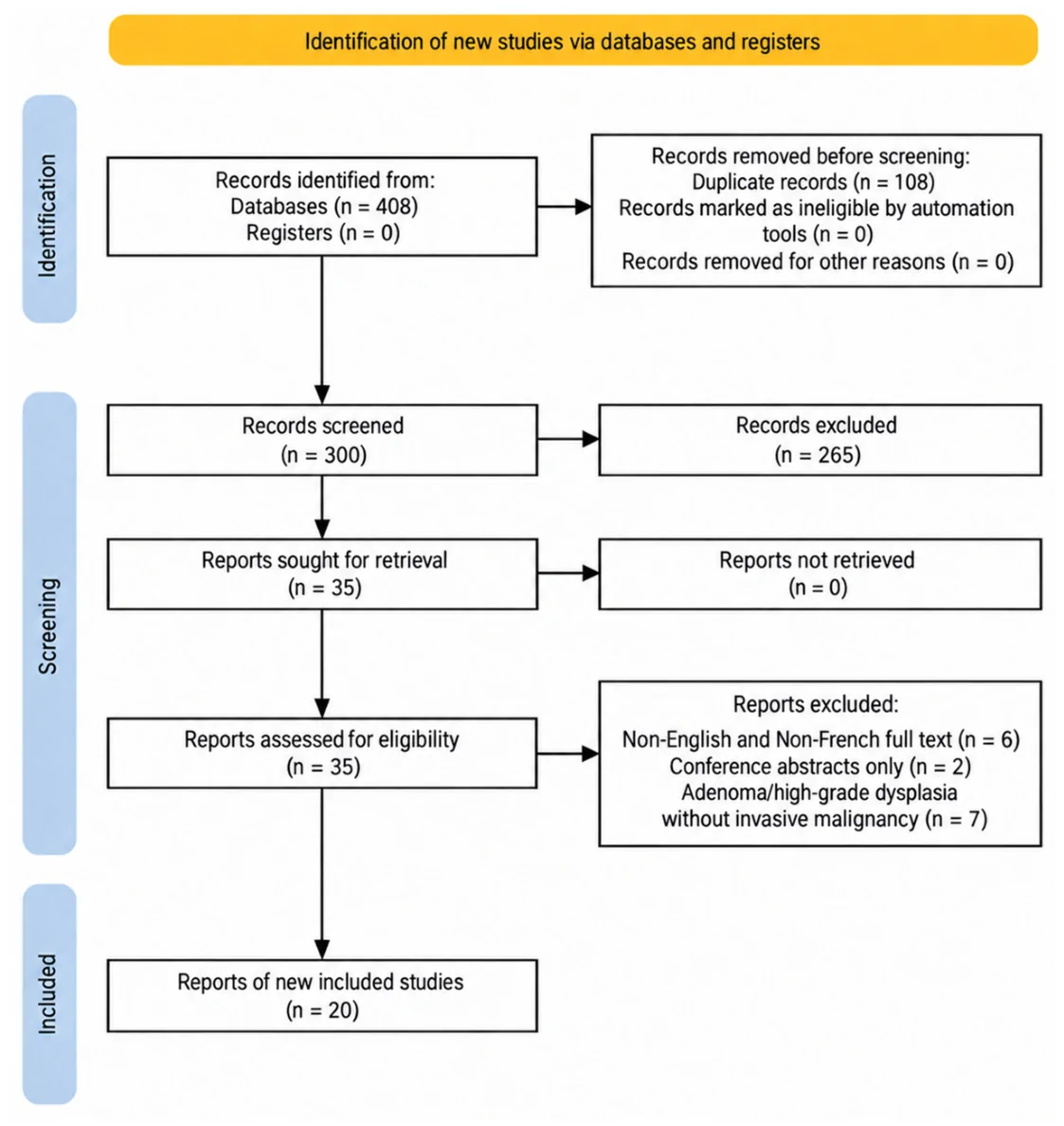
PRISMA 2020 flow diagram showing identification, screening, eligibility assessment, and inclusion of studies in the systematic review.

The initial search identified 408 (224 on PubMed and 184 on Web of Science) articles. After duplicate removal, 300 articles were screened for eligibility by two independent investigators (IM and GG) with disagreements solved by consensus with a third investigator (ILAI). Only English- and French-language case reports of malignancies on colon interposition in the esophagus were included. The language restriction was applied because these were the languages in which the reviewers could reliably assess the full text and extract detailed clinical data without translation-related uncertainty. After abstract screening, 35 articles remained. Full-text screening resulted in removing 15 further articles (6 with full text in another language, 2 were only conference abstracts, and 7 reported adenoma/high-grade dysplasia only), 20 articles were included in the final review. For each report, we recorded age and sex, initial indication for esophagocoloplasty, interposed colonic segment, interval between esophagocoloplasty and cancer diagnosis, histological tumor type metastatic presentation at diagnosis, systemic therapy, radiotherapy, surgical intervention, reconstruction or management after treatment, and outcome or follow-up. The characteristics of the included cases are summarized in Table 1. The systematic review was conducted and reported in accordance with the PRISMA 2020 statement. The study-selection process is summarized in a PRISMA 2020 flow diagram (Figure 1). No review protocol was prospectively registered in PROSPERO.

The methodological quality of the included case reports was assessed using the JBI Critical Appraisal Checklist for Case Reports. The checklist evaluates eight domains relating to patient description, clinical history, presentation, diagnostic assessment, treatment, post-intervention condition, adverse events, and takeaway lessons. Each item was rated as Yes, No, Unclear, or Not Applicable. Two reviewers independently (IM and GG) performed the appraisal, with disagreements resolved solved by consensus with a third investigator (ILAI). The complete appraisal is provided in Table A1.

## 3. Results

### 3.1. Case Presentation

We present the case of a 67-year-old man that had ingested caustic substances during a suicide attempt and was treated by isoperistaltic left coloesophagoplasty in 1979.

**Figure 2:**
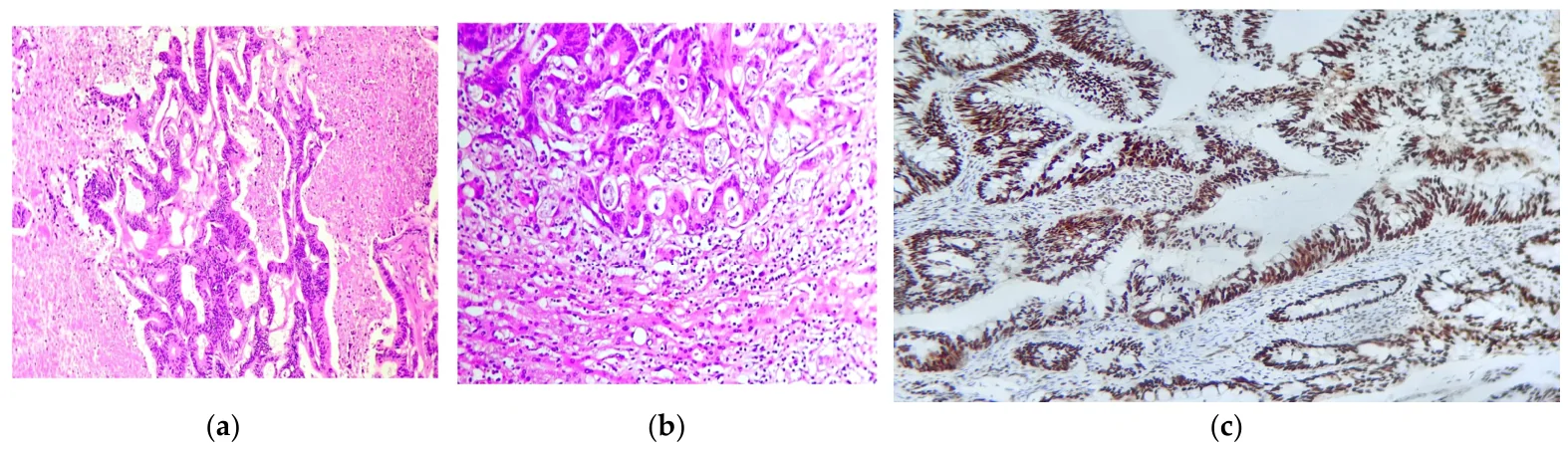
(**a**,**b**): Representative H&E-stained section (×200) of the liver metastasis from moderately differentiated (G2) colonic adenocarcinoma showing infiltrative glandular structures with moderate cytologic atypia, luminal dirty necrosis, and a prominent desmoplastic stromal reaction replacing the hepatic parenchyma; (**c**): SATB2 immunohistochemical staining (×200) of the hepatic metastasis demonstrating strong, diffuse nuclear positivity in the tumor cells, consistent with metastatic colonic adenocarcinoma.

In 2023, during evaluation in the context of a prior right nephroureterectomy for urothelial carcinoma, stage 1, abdominal imaging identified suspicious hepatic lesions. Abdominal magnetic resonance imaging (MRI) performed in August 2023 described two hepatic lesions located in the right liver, in segments VIII and VI, with imaging characteristics suggestive of secondary tumor involvement. Histopathological and immunohistochemical assessment of hepatic tissue, reported in October 2023, demonstrated metastatic moderately differentiated adenocarcinoma, grade 2 (Figure 2). The tumor cells expressed SATB2 (Figure 2c) and CK20 and were negative for CK7. Mismatch repair protein expression was retained, supporting a proficient mismatch repair status, and the Ki-67 proliferation index was reported as 90%. Specific antibody clones and manufacturers were not available in the original pathology records. Molecular testing identified a KRAS exon 2 mutation. Additional molecular results, including NRAS, BRAF V600E, HER2, and NTRK status, were not performed at the time.

Whole-body positron emission tomography–computed tomography (PET-CT) in November 2023 further characterized the disease, showing a metabolically active parietal thickening of the colonic esophagoplasty segment at the anastomosis, extending cranio-caudally over approximately 68 mm with increased 18F-fluorodeoxyglucose (FDG) uptake.

**Figure 3:**
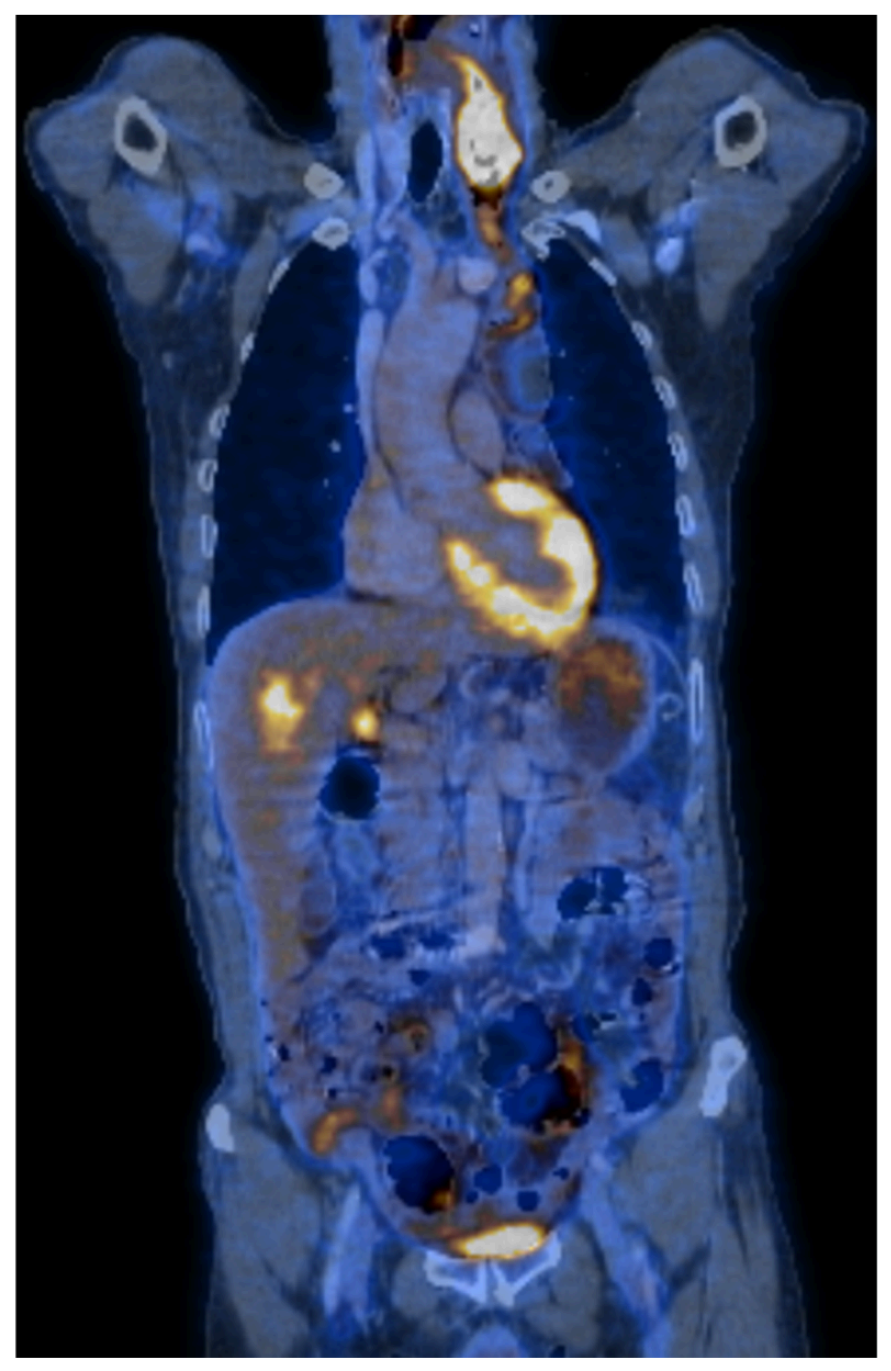
Whole-body PET-CT demonstrating multiple metabolically active hepatic lesions and a cervical lesion with increased FDG uptake.

**Figure 4:**
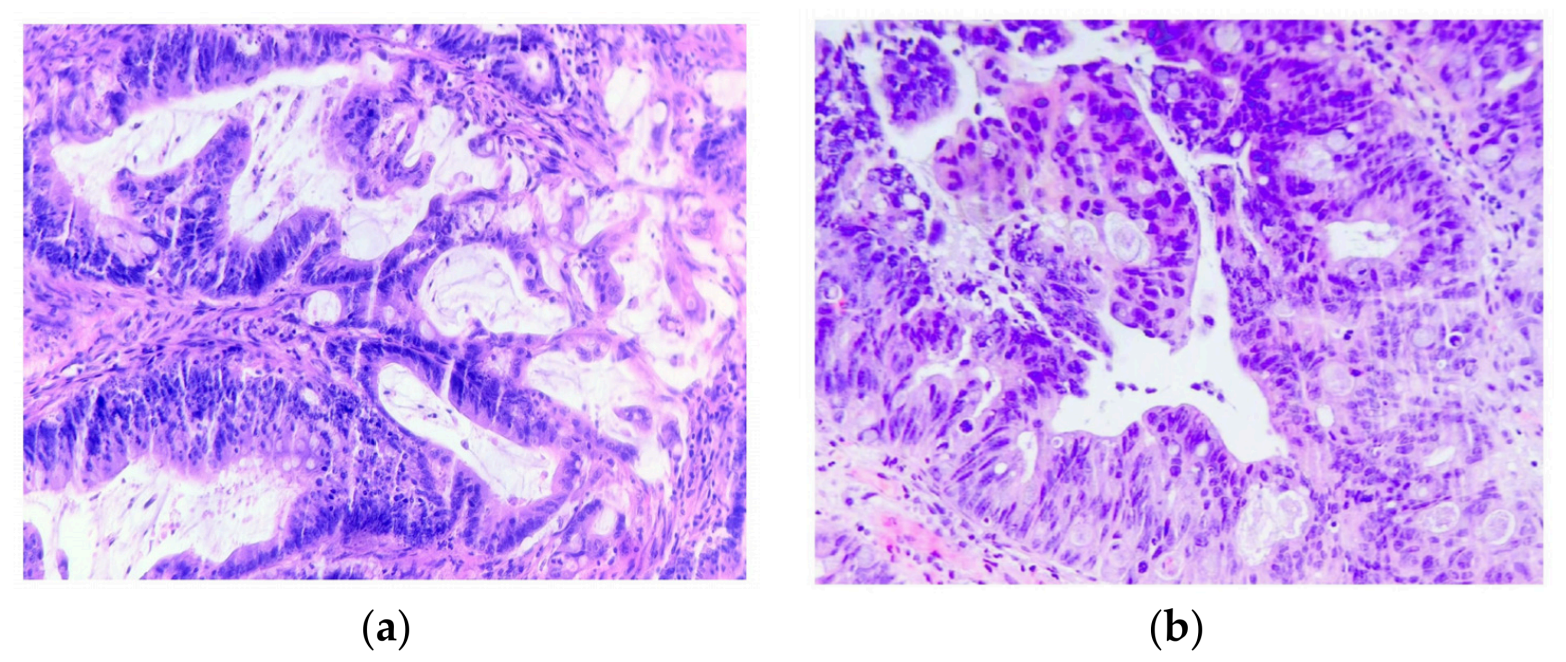
(**a**,**b**): Representative H&E-stained sections (×200) of the biopsy from the esophageal tumoral mass showing moderately differentiated (G2) adenocarcinoma with irregular infiltrative glands, moderate cytologic atypia, dirty necrosis, and desmoplastic stromal reaction.

Multiple metabolically active hepatic lesions were also present (Figure 3). No metabolically active cervical, axillary, mediastinal, or abdominopelvic lymphadenopathy or pathological pulmonary uptake was reported at that time. The patient was assessed by upper endoscopy, and a biopsy of the tumoral mass at the cervical level of the interposed colonic graft was subsequently performed. Histopathological examination showed a moderately differentiated adenocarcinoma, grade 2 (Figure 4).

The diagnosis was therefore metastatic left colon adenocarcinoma arising in the interposed colonic graft, initially staged cTxN0M1a with hepatic metastases, grade 2, proficient mismatch repair, and KRAS-mutated disease.

The case was discussed by the multidisciplinary tumor board in December 2023, and systemic treatment with FOLFOX plus bevacizumab was initiated, continuing until July 2024. Abdominal and pelvic MRI in April 2024 documented oncological regression compared with the previous evaluation, with persistence of the two hepatic lesions and hemicircumferential thickening of the colonic esophagoplasty segment. Thoracic CT the same month showed no pulmonary lesions with proliferative or secondary tumor characteristics, although small micronodules were noted for imaging surveillance.

In June 2024, given the patient’s cardiac comorbidity and sensory neuropathy, the multidisciplinary team de-escalated treatment to fluorouracil and folinic acid/leucovorin (FUFOL) plus bevacizumab. Imaging in August 2024 (brain MRI with abdominal and pelvic assessment, and thoracic CT) described a stationary oncological appearance: no secondary cerebral lesions, hepatic lesions numerically and dimensionally stable, and unchanged thickening of the colonic esophagoplasty segment.

MRI of the abdomen and pelvis in February 2025 showed stable hepatic lesions and persistent hemicircumferential thickening of the colonic esophagoplasty segment. However, contrast-enhanced cervical and thoracic CT later that month demonstrated a proliferative lesion in the inferior cervical region of the same segment, measuring approximately 4.2 cm × 4.2 cm × 5.4 cm, with heterogeneous enhancement, apparent erasure of the separation plane with the sternocleidomastoid muscle, anterior displacement of the left thyroid lobe, and direct relation with the common carotid artery and the compressed left internal jugular vein. Thoracic CT also identified a newly developed 0.6 cm iodophilic pulmonary nodule in the anterior segment of the right upper lobe, considered suspicious and requiring monitoring.

PET-CT in March 2025 confirmed metabolically active cervical progression. It described a necrotic, FDG-avid left laterocervical tumor mass around the anastomosis, with a maximum standardized uptake value of 16.55, measuring approximately 3 cm × 4 cm and extending longitudinally at least 6 cm. The lesion partially surrounded the left jugulocarotid vascular bundle, extended supraclavicularly, and followed the course of the colonic graft. Increased FDG uptake was also noted in the inferior portion of the colonic plasty at the gastric anastomosis, metabolically active hepatic lesions persisted, and additional metabolically active peritoneal lesions were described. Systemic therapy was therefore changed to folinic acid, fluorouracil, and irinotecan (FOLFIRI) plus bevacizumab in March 2025.

**Figure 5:**
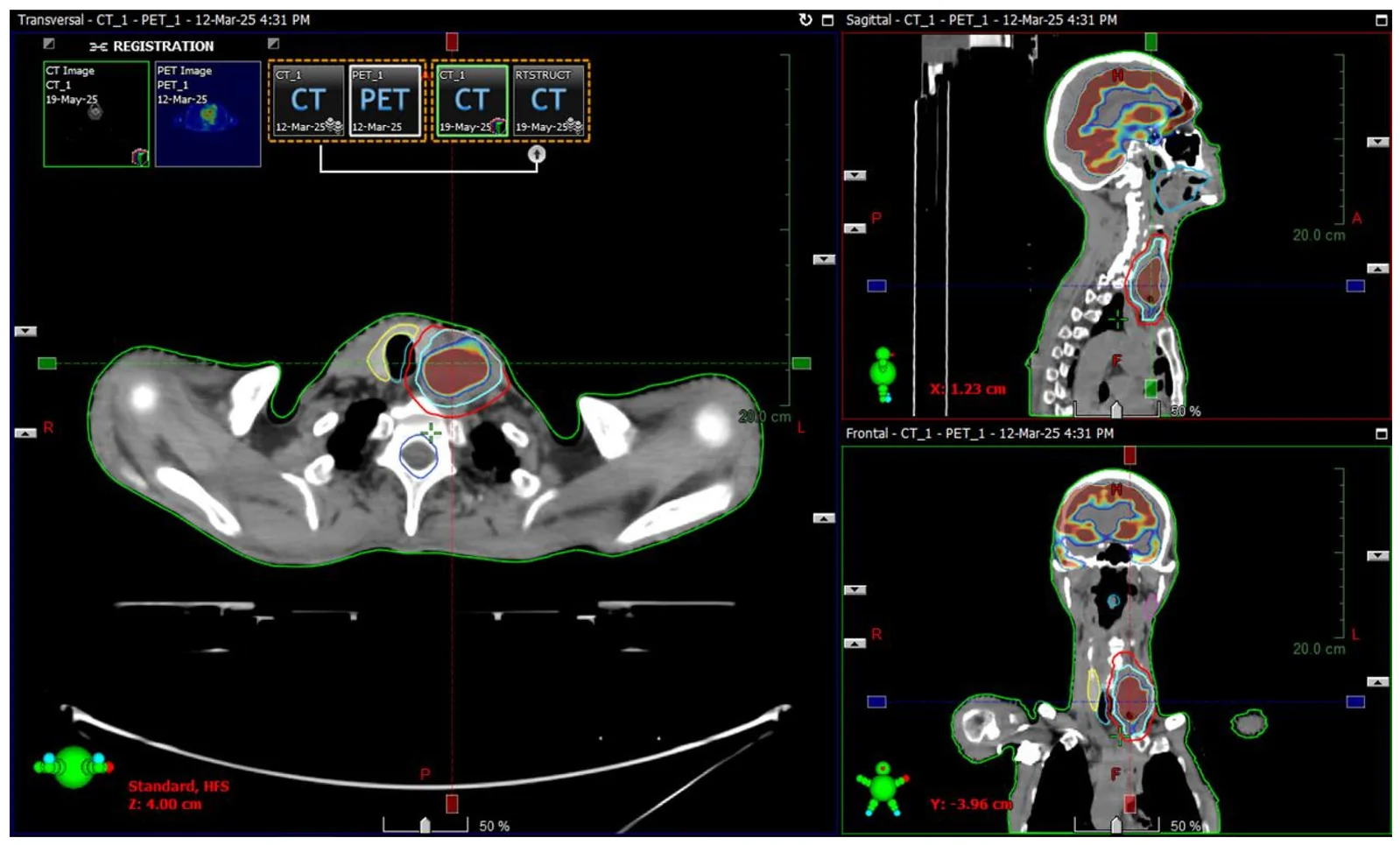
PET-CT–guided target volume delineation for cervical IMRT. The clinical target volume is shown in blue and the planning target volume in red.

Two months later the patient presented with dysphagia for solid foods. The case was again discussed in the oncology board in May 2025, when, considering the locoregional impact of tumor progression and a Karnofsky Performance status of 70, radiotherapy was recommended. Systemic treatment was stopped and the patient underwent cervical intensity-modulated radiotherapy (IMRT) between May and July 2025, to a total dose of 50.4 Gy in 28 fractions with daily cone-beam CT (CBCT) image guidance. Treatment was delivered using a TrueBeam linear accelerator (Varian Medical Systems, Palo Alto, CA, USA) and planned using the Eclipse treatment planning system, version 17.0 (Varian Medical Systems, Palo Alto, CA, USA). The target volume included the tumor mass within the interposed colonic segment at the cervical level (Figure 5). Radiotherapy was selected to address the progressing cervical lesion and reduce local tumor burden in a complex anatomical region involving the colonic graft and adjacent cervical vascular and soft-tissue structures. After 20 fractions, weight loss and tumor shrinkage produced anatomical changes within the radiation field, requiring repeat simulation; an adaptive plan was generated to maintain target coverage and improve protection of adjacent organs at risk. The patient developed grade I (RTOG scale) acute radiation dermatitis and esophagitis.

Post-radiotherapy contrast-enhanced cervical and thoracic CT in July 2025 showed mild regression of the inferior cervical lesion within the colonic esophagoplasty segment, now measuring approximately 3.5 cm × 3.6 cm × 5.1 cm. No fistulous tract or extravasation of oral contrast from the digestive lumen was identified, although contrast administration was limited by the gastrostomy and reduced oral intake. Despite the cervical response, systemic progression was documented, with numerical and dimensional progression of pulmonary nodules and progression of hepatic disease. As systemic therapy had not been given between May and July 2025, FOLFIRI plus bevacizumab was continued thereafter.

**Figure 6:**
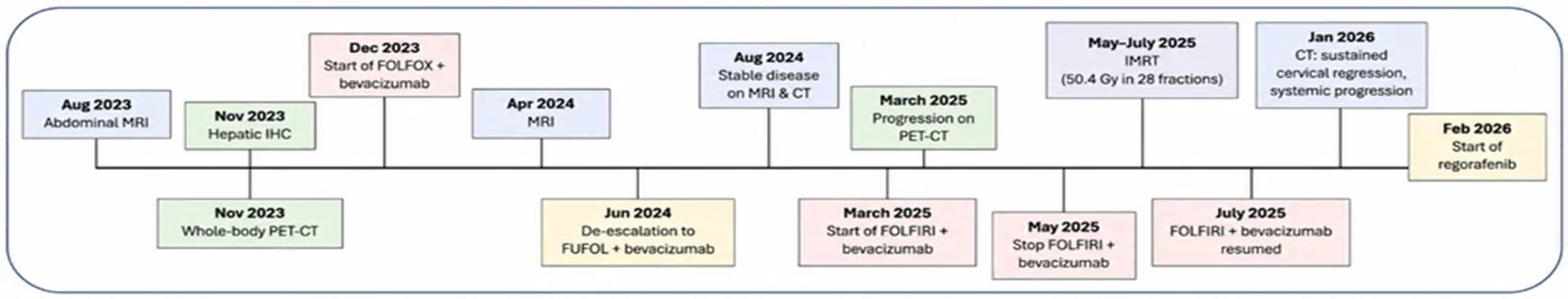
Timeline of the diagnostic work-up, systemic treatment, radiotherapy, and disease evolution in the present case. Blue boxes indicate diagnostic investigations and imaging assessments, pink boxes indicate systemic treatment and treatment modifications, green boxes indicate PET-CT/IHC-related diagnostic assessments, and yellow boxes indicate treatment de-escalation or later-line therapy.

Further CT evaluation in January 2026 demonstrated additional moderate regression of the cervical tumor, now measuring approximately 2 cm × 2 cm × 5.1 cm. The lesion remained heterogeneous, with necrotic areas, persistent loss of the separation plane with the sternocleidomastoid muscle, mass effect on the left thyroid lobe, and direct relation with the common carotid artery and the mildly compressed left internal jugular vein. At the same time, systemic disease progression was evident, with hepatic metastases and intrahepatic bile duct dilatation in progression, pulmonary secondary nodules including one newly developed lesion, and partial venous thromboses involving the left subclavian vein and left brachiocephalic venous trunk. FOLFIRI plus bevacizumab was last documented in December 2025 and, given further metastatic progression, treatment was switched to regorafenib in February 2026. At discharge from the oncology department that month, the patient’s condition was described as improved, with ongoing oncological follow-up recommended. The complete timeline of the diagnostic work-up, systemic treatment, radiotherapy, and disease evolution is summarized in Figure 6.

### 3.2. Literature Review

Malignancies arising within an interposed colonic segment after esophagocoloplasty appears to be an uncommon late complication, with the available literature consisting mainly of isolated case reports. Among the 20 cases included in the review, the median age at diagnosis was 61.5 years (IQR 56–67.75 years; range 33–76 years). Sex was reported in 19 of 20 cases; 11/19 patients (57.9%) were male and 8/19 (42.1%) were female. The present patient, aged 67 years, therefore falls within the reported age range.

The original indication for reconstruction was a benign esophageal injury or stricture in 12/20 cases (60%), including caustic, corrosive, alkaline, or lye-induced injury in 11/20 cases (55%). Previous esophageal or gastroesophageal malignancy accounted for 4/20 cases (20%), congenital esophageal atresia for 3/20 cases (15%), and severe reflux-related esophago-bronchial fistula for 1/20 cases (5%). Histologically, adenocarcinoma was the predominant tumor type, reported in 17/20 cases (85%), while squamous cell carcinoma occurred in 2/20 cases (10%) and gastrointestinal stromal tumor (GIST) in 1/20 cases (5%). The median interval between esophagocoloplasty and diagnosis of the reported neoplasm was 20 years (IQR 14.5–40.5 years; range 2–60 years). In 6/20 cases (30%), the neoplasm was diagnosed 40 years or more after reconstruction. Metastatic status at diagnosis was reported in 19/20 cases; 5/19 patients (26.3%) presented with metastatic disease, whereas 14/19 (73.7%) had no reported metastases at initial diagnosis.

Treatment strategies depended on disease extent. In localized or resectable cases, reported interventions included resection of the tumor-bearing colonic conduit or graft, followed by gastric, jejunocolic, or esophagogastric reconstruction. Endoscopic treatment was reported for early mucosal, polyp-associated lesions, including polypectomy, endoscopic mucosal resection, endoscopic submucosal dissection, and balloon dilatation when benign stricture was also present. When reported, systemic therapy included 5-fluorouracil-based regimens, FOLFOX or modified FOLFOX6, FOLFIRI-based treatment, CAPOX, capecitabine, palliative chemotherapy, and pembrolizumab. In the present case, systemic treatment included FOLFOX plus bevacizumab, fluorouracil/folinic acid plus bevacizumab, FOLFIRI plus bevacizumab, and later regorafenib.

Radiotherapy was inconsistently described. It was often not administered, and when administered details were not reported; one case received palliative external-beam radiotherapy and died after the 16th session [9], another reported palliative radiotherapy [3], and one had proposed neoadjuvant radiotherapy that was refused [17]. The present case therefore adds detailed information on cervical IMRT. Outcomes were heterogeneous, ranging from death at 2 months after disease discovery [3] to 60 months of disease-free survival [11]. Advanced, locally progressive or metastatic cases were frequently recommended palliative care [3,7], and suffered treatment interruptions [2,7,14]. In the present case, cervical regression after radiotherapy contrasted with subsequent systemic progression, illustrating the need to address both locoregional and metastatic disease.

## 4. Discussion

Malignancies arising in a colonic interposition graft after esophagocoloplasty is an uncommon late complication and becomes particularly challenging when the tumor develops in the cervical portion of the reconstructed gastrointestinal tract. In the present case we describe a 67-year-old man with previous caustic ingestion during a suicide attempt, followed by isoperistaltic coloesophagoplasty in 1979, who developed a colon-type adenocarcinoma in the interposed colonic segment approximately 44 years later. The clinical importance of this case lies not only in the long latency, but also in the discordance between tumor biology and tumor location. This distinction had direct implications for systemic treatment selection and radiotherapy planning. The novelty of our case stems from the detailed radiotherapy regimen description, as no other article we found during our systematic review presented it. Previous literature analyses [5,6] and our review of the literature confirm that malignant transformation in colon interposition grafts is rare, usually described as isolated cases rather than as a well-defined oncological entity [2,3,7,19,25].

The development of malignancies in an interposed colon segment has been reported decades after reconstruction for benign esophageal disease, including caustic strictures. We found that latency might vary from 2 to 60 years, similar to Schraps, who described cases of colorectal adenocarcinoma arising 57 years after esophageal substitution in infancy [19]. The pathogenesis remains uncertain. Proposed mechanisms include chronic exposure of colonic mucosa to acid or bile reflux, food stasis, inflammation, altered luminal microbiota, chronic irritation, and the intrinsic adenoma–carcinoma sequence that may occur in colonic mucosa regardless of its transposed location [2,3,7,25]. These mechanisms are biologically plausible, but causality cannot be inferred from the available evidence because published data consist mainly of case reports and small narrative reviews [2,3,7,19,25].

In the present case, the long interval between coloesophagoplasty and diagnosis is consistent with the delayed presentation described in the literature. This supports the need for long-term endoscopic surveillance of colon grafts, although no universally accepted protocol exists. Some authors have proposed follow-up endoscopy of the interposed colon beginning after reconstruction and repeated at intervals of 2–3 years, while others suggest adapting surveillance principles used for the native colon to the graft, particularly because neoplastic lesions in the graft may follow similar biological pathways; however, no standardized surveillance protocol has been established [2,25].

The key conceptual point of this case is that treatment should be guided by the organ and tissue of origin rather than by the anatomical site of the tumor. The irradiated tumor-bearing structure was not the native cervical esophagus, but an interposed colonic segment; the immunophenotype (CK20-positive, SATB2-positive, CK7-negative, pMMR, KRAS exon 2 mutation) and the diagnosis of metastatic colon-type adenocarcinoma supported management according to colorectal biology, even though the cervical location imposed technical constraints typical of head-and-neck or upper mediastinal irradiation, including the proximity of the carotid–jugular vascular bundle, thyroid, laryngeal structures, spinal cord, skin, and reconstructed digestive tract. Constantin et al. make a similar argument, stating that treatment of adenocarcinoma arising in a colon graft does not fundamentally differ from treatment of colon cancer “in situ” up to a point, but must be adapted to staging and to the new topography of the interposed organ [25]. Given this metastatic disease at diagnosis and the colorectal immunophenotype described above, first-line systemic therapy was prioritized by the multidisciplinary team. This approach is concordant with contemporary international recommendations for unresectable metastatic colorectal cancer, which list fluoropyrimidine–oxaliplatin-based regimens such as FOLFOX, with or without bevacizumab, among recommended systemic therapy options for advanced or metastatic colon cancer [26,27]. This choice aligns with the literature we analyzed, where most cases were treated with a fluoropyrimidine-based regimen [2,7,14,15,19].

Over the following two years, systemic therapy was adapted sequentially to comorbidity and to disease trajectory rather than to the anatomical location of the mass. FOLFOX plus bevacizumab was de-escalated to FUFOL plus bevacizumab because of cardiac comorbidity and sensory neuropathy rather than disease progression; following subsequent locoregional and peritoneal progression, treatment was changed to FOLFIRI plus bevacizumab, consistent with second-line sequencing after prior oxaliplatin exposure. Cervical radiotherapy was then added for locoregional control, producing tumor regression while FOLFIRI plus bevacizumab was continued; when systemic disease later progressed with hepatic and pulmonary involvement, treatment was switched to regorafenib, consistent with later-line therapy after progression on fluoropyrimidine-, oxaliplatin-, and irinotecan-based regimens. This sequence reinforces the central message of the case: the therapeutic framework followed colorectal tumor biology and international colorectal cancer guidelines, not the anatomical location of the tumor.

Radiotherapy was considered because the cervical lesion progressed in a clinically sensitive region, with local mass effect and vascular compression, while systemic therapy alone was no longer providing adequate locoregional control. The target volume included the tumor mass within the interposed colonic segment at the cervical level. The gross tumor volume (GTV) was delineated based on the available imaging. A 5 mm isotropic margin was added to generate the clinical target volume (CTV), followed by manual editing to respect anatomical boundaries and exclude uninvolved bone, air spaces, and uninvolved adjacent muscle. The planning target volume (PTV) was generated using a further 5 mm isotropic expansion of the CTV. Both the CTV and PTV were cropped 3 mm from the external body contour. The patient underwent cervical IMRT to 50.4 Gy in 28 fractions delivered with daily cone-beam CT image guidance to the tumor mass; this conventional-fractionation regimen, extrapolated from colorectal adenocarcinoma radiotherapy experience, was chosen to balance locoregional tumor control against toxicity to adjacent cervical structures, while planning, immobilization, image guidance, and adaptive replanning addressed the risks of treating a mobile, surgically altered conduit adjacent to cervical vessels, thyroid, airway, and soft tissues. This dose selection deserves comment: in conventional colorectal practice, radiotherapy is central to rectal cancer management but has a much more limited role in typical colon cancer, where surgery and systemic therapy predominate. Our choice of regimen amounted to a local control of at least 8 months. The published reports of malignancies in colonic interpositions rarely provide directly comparable radiotherapy details: one reported late-stage case received chemotherapy and radiotherapy without sufficient accessible dose-fractionation information for meaningful comparison [3].

This case provides a clear long-term clinical chronology, documents the rare relationship between prior esophageal substitution and later graft malignancy, includes histopathology and immunohistochemistry supporting colorectal origin, reports key molecular information, and describes multidisciplinary adaptation of systemic therapy and radiotherapy, including adaptive replanning in response to treatment-related anatomical change in a complex cervical field.

However, this is a single case, so no generalizable efficacy or safety conclusions can be drawn. The available documents do not report complete endoscopic surveillance history, patient perspective, detailed radiotherapy toxicity grading over time, performance status, or long-term follow-up after regorafenib. Molecular data were incomplete for contemporary metastatic colorectal cancer decision-making because NRAS, BRAF V600E, HER2, NTRK, and broader next-generation sequencing results were not reported.

Finally, the literature base for radiotherapy in cervical colon-graft malignancies remains too limited to define a standard approach; treatment was therefore based on colorectal histology, metastatic colorectal cancer evidence, local anatomy, and multidisciplinary judgment.

Regarding the limitations of the literature review, the search may not have been exhaustive. The use of restrictive NOT terms may have reduced the sensitivity of the search strategy and could have resulted in the omission of some eligible reports in which previous esophageal or gastric malignancy was also mentioned. These exclusion terms were nevertheless used to improve search precision by reducing the large number of records concerning primary gastroesophageal malignancies treated with procedures other than esophagocoloplasty. The restriction to English- and French-language publications may have resulted in the exclusion of otherwise eligible reports published in other languages.

## 5. Conclusions

Malignancies arising in an interposed colonic segment after esophagocoloplasty is an uncommon but clinically important late complication. The present case demonstrates metastatic colon adenocarcinoma developing approximately 44 years after isoperistaltic coloesophagoplasty and later progressing within the cervical portion of the graft. It emphasizes that an interposed colon should not be considered biologically exempt from colorectal neoplasia.

Management required integration of pathology, immunohistochemistry, molecular profiling, cross-sectional and functional imaging and multidisciplinary judgment. Systemic treatment followed metastatic colorectal cancer principles, whereas radiotherapy required adaptation to cervical anatomy, daily image guidance, and adaptive replanning. This well-documented report adds to the limited literature on graft-associated colon adenocarcinoma and describes a complete radiation regimen using modern techniques. IMRT was associated with radiographic regression and sustained local control during the reported follow-up, despite progression of systemic disease.

## Figures and Tables

**Table 1:** Summary of published case reports describing malignancies of the interposed colon after esophagocoloplasty.

Author/ Year	Age (Years)/ Sex (F/M)	Histological Tumor Type	Indication for Esophagocoloplasty	Interposed Colonic Segment	Interval Until Cancer Diagnosis	Metastases at Diagnosis (Yes + Location/No)	Systemic Therapy	Radiotherapy Details	Surgical Intervention	Reconstruction/Management After Treatment	Outcome/Follow-Up
Aryal 2013 [7]	60/M	Adenocarcinoma	Severe esophageal stricture secondary to caustic lye ingestion	Right colon	30 years	Yes, liver	5-fluorouracil chemotherapy	Not reported	No	PEG for decompression and jejunal tube for feeding; ultimately palliative care chosen	Progression of disease with gastric outlet obstruction; patient chose palliative care
Badshah 2014 [8]	47/M	GIST	Caustic alkali (NaOH/lye) ingestion	Not reported	~20 years	Yes, liver	Imatinib 400 mg orally daily	No	No	Managed medically with imatinib, pain control, and a bowel regimen; no surgical resection or reconstruction	Doing well at 3-month follow-up; follow-up CT showed considerable tumor shrinkage
Barbosa 2018 [2]	70/F	Adenocarcinoma	Severe esophageal stricture after caustic ingestion	Left colon	19 years	No	Planned neoadjuvant FOLFIRI plus cetuximab (400 mg/m^2^) for six cycles; after progression, palliative chemotherapy was started	Not reported	Yes	Surgical gastrostomy; planned removal of colonic graft and definitive salivary fistula was not performed after progression; palliative chemotherapy after disease progression	Disease progression after six cycles; died 6 months after initial diagnosis from severe respiratory failure caused by pulmonary thromboembolism
Bazine 2014 [9]	33/M	Squamous cell carcinoma	Accidental caustic burn of the esophagus	Not reported	13 years	No	No	External-beam radiotherapy (palliative); patient died during the 16th session	No	Feeding jejunostomy and palliative external radiotherapy	Died during the 16th radiotherapy session due to deterioration of general condition
Cheng 2015 [10]	40/F	Adenocarcinoma	Corrosive esophageal injury after attempted suicide	Right colon	15 years	No	Not reported	Not reported	Not reported	Not reported	Died 4 months after admission from nosocomial infections
De Moura 2018 [3]	63/F	Adenocarcinoma	Caustic soda ingestion with corrosive esophageal injury/benign stricture; refractory to dilation before subtotal esophagectomy	Not reported	8 years	Yes, bilateral lung metastases	Palliative chemotherapy	Palliative radiotherapy	No	Palliative chemotherapy and radiotherapy	Died 2 months after diagnosis
Iascone 2019 [11]	65/F	Adenocarcinoma	Lye-induced (caustic) multiple esophageal strictures (corrosive ingestion at age 6); esophagectomy at age 28	Left colon	35 years	No	FOLFOX, 6 cycles	No	No (tumor removed endoscopically)	Complete endoscopic submucosal removal of the tumor; adjuvant FOLFOX; periodic endoscopic and CT surveillance	Disease-free with good lifestyle and regular diet 60 months after tumor removal
Kia 2010 [12]	76/M	Adenocarcinoma	Early-stage esophageal adenocarcinoma	Not reported	15 years	No	No	Not reported	No (treated with endoscopic mucosal resection/polypectomy)	Endoscopic mucosal resection (snare cautery polypectomy, saline-lift assisted) of interposed-colon polyps; PEG tube removed	Dysphagia resolved; tolerated a regular diet; all resection margins free of tumor (follow-up duration not reported)
Kim 2012 [13]	70/F	Adenocarcinoma	Total esophagectomy for esophageal stricture due to alkaline corrosive injury	Right colon	47 years	No	5-fluorouracil (400 mg/m^2^) + oxaliplatin (85 mg/m^2^) combination chemotherapy	Not reported	No (patient declined surgical treatment)	Palliative combination chemotherapy (5-FU + oxaliplatin); patient declined surgery	Partial response after 6 cycles; died of pneumonia during admission for the 8th cycle, 6 months after initial diagnosis
Kim 2025 [14]	70/F	Adenocarcinoma	Caustic ingestion-induced stricture	Not reported	60 years	Yes, multiple hepatic metastases	1 cycle 5-fluorouracil, leucovorin, and irinotecan; 8 cycles combination chemotherapy with bevacizumab	Not reported	No	Self-expandable metal stent insertion for progressive dysphagia; palliative systemic treatment and CT surveillance every 3 months	Partial radiological response after 3 months, with reduction of the primary tumor and hepatic metastases; chemotherapy discontinued after 6 months due to deterioration of general condition
Kohli 2019 [15]	47/F	Adenocarcinoma	Gastroesophageal junction carcinoma	Ileocolonic conduit	4.5 years	No	Adjuvant single-agent capecitabine	Not reported	Yes	Resection of tumor-bearing colonic conduit with adjacent diaphragm, liver wedge excision, and left adrenalectomy; jejunocolic anastomosis refashioned using a transoral circular stapler	Uneventful postoperative period; last follow-up at 30 months postoperatively
Kröner 2015 [16]	67/M	Adenocarcinoma	Esophageal metastatic adenocarcinoma	Not reported	10 years	Not reported	Not reported	Not reported	Not reported	Endoscopic dual self-expanding metal stent placement; Fully covered 12 cm esophageal stent at the cologastric anastomosis and uncovered 10 cm colonic stent in stent-in-stent fashion to anchor it	No complications; symptoms improved; discharged home the same day
Maouni 2025 [17]	59/M	Squamous cell carcinoma	Unintentional caustic ingestion	Left colon	29 years	No	No	Neoadjuvant radiotherapy proposed but not administered, patient refused	No (surgery planned but patient refused)	Planned jejunostomy, neoadjuvant radiotherapy, then graft resection; patient refused all proposed management	Patient refused treatment and was lost to follow-up
Sallum 2014 [18]	53/F	Adenocarcinoma	Congenital esophageal atresia	Not reported	42 years	No	Yes (started after lung metastases developed)	Not reported	Yes	Resection of the transposed colon; transhiatal esophagectomy of the atresic esophagus; resection of invaded left innominate vein; greater-curvature gastric conduit pulled up; postoperative partial gastrectomy for ischemia of the proximal stomach	Discharged after 128 days; developed lung metastases, started chemotherapy, and died 9 months after surgery due to pneumonia
Schraps 2024 [19]	57/NR	Adenocarcinoma	Esophageal atresia in infancy	Not reported	57 years	No	Adjuvant CAPOX started approximately 2 months after surgery; after bipulmonary metastases, switched to palliative FOLFIRI; later third-line therapy not specified	Not reported	Yes	Thoracoabdominal esophagectomy with en bloc resection of colonic interposition and adjacent healthy esophagus; atypical right upper-lobe lung resection after intraoperative lung injury; gastric pull-up reconstruction	First follow-up in July 2023 revealed bipulmonary metastases; pulmonary progression continued despite systemic therapy
Shersher 2011 [20]	60/M	Adenocarcinoma	Benign esophageal stricture	Not reported	40 years	No	Not reported	Not reported	Yes	En bloc resection of the tumor and colon interposition segment; Ivor Lewis esophagectomy with end-to-end esophagogastric anastomosis; feeding jejunostomy	Primary esophagogastric anastomosis; postoperative chylothorax treated with thoracic duct ligation and small anastomotic leak treated with stent placement; after stent removal, leak resolved; patient tolerated a regular diet and reported significant functional improvement
Spitali 2012 [21]	66/M	Adenocarcinoma	Total esophagectomy for recurrent adenocarcinoma derived from Barrett esophagus	Right colon	2 years	No	Not reported	Not reported	Yes	Right thoracotomy with ileocecal resection and end-to-end anastomosis; end-to-end anastomosis; marginal arcade artery preserved to prevent necrosis of the proximal ileal segment	Disease-free at 24 months
Takasugi 2019 [22]	70/M	Adenocarcinoma	Esophago-bronchial fistula due to severe reflux esophagitis	Left colon	20 years	No	Neoadjuvant modified FOLFOX6 (l-leucovorin/5-FU/oxaliplatin) x 6 courses	Not reported	Yes, radical total resection of the colonic graft and cervical esophagus with lymph node dissection (median sternotomy)	Resection of cervical esophagus and total colonic graft with lymph node dissection; gastric tube reconstruction via retrosternal route (postoperative left recurrent laryngeal nerve palsy)	Local and pleural recurrence 6 months after resection; disease-free survival 6 months, postoperative survival 17 months, overall survival 21 months
Tranchart 2014 [23]	66/M	Adenocarcinoma	Caustic ingestion (corrosive esophageal injury)	Right colon	19 years	Yes, liver and mediastinum	Palliative FOLFOX chemotherapy	Not reported	No	Palliative FOLFOX chemotherapy	Died 3 months after diagnosis from liver failure
Yancey 2025 [24]	59/M	Adenocarcinoma	Esophageal atresia in infancy	Right colon	59 years	No	Pembrolizumab immunotherapy every 3 weeks, with option to transition to every 6 weeks, planned up to 2 years	Not reported	No	Active treatment with pembrolizumab plus interval endoscopic and imaging surveillance every 4 months	Remained under active treatment; response/progression beyond active surveillance not reported

FOLFOX—folinic acid + fluorouracil + oxaliplatin; CAPOX—capecitabine + oxaliplatin; FOLFIRI—folinic acid + fluorouracil + irinotecan; PEG—percutaneous endoscopic gastrostomy.

## Data Availability

No new data were created or analyzed in this study. Data sharing is not applicable to this article.

## Appendix A

**Table A1:** Methodological quality assessment of included case reports using the JBI Critical Appraisal Checklist.

Article	Q1	Q2	Q3	Q4	Q5	Q6	Q7	Q8
Aryal 2013 [7]	Yes	Yes	Yes	Yes	Yes	Yes	Unclear	Yes
Badshah 2014 [8]	Yes	Yes	Yes	Yes	Yes	Yes	No	Yes
Barbosa 2018 [2]	Yes	Yes	Yes	Yes	Yes	Yes	Yes	Yes
Bazine 2014 [9]	Yes	Yes	Yes	Yes	Yes	Yes	Yes	Yes
Cheng 2015 [10]	Yes	Yes	Yes	Yes	No	N/A	Yes	Yes
De Moura 2018 [3]	Yes	Yes	Yes	Yes	Unclear	Yes	Yes	Yes
Iascone 2019 [11]	Yes	Yes	Yes	Yes	Yes	Yes	No	Yes
Kia 2010 [12]	Yes	Yes	Yes	Yes	Yes	Yes	No	Yes
Kim 2012 [13]	Yes	Yes	Yes	Yes	Yes	Yes	Yes	Yes
Kim 2025 [14]	Yes	Yes	Yes	Yes	Yes	Yes	Yes	Yes
Kohli 2019 [15]	Yes	Yes	Yes	Yes	Yes	Yes	No	Yes
Kröner 2015 [16]	Yes	Yes	Yes	Yes	Yes	Yes	No	Yes
Maouni 2025 [17]	Yes	Yes	Yes	Yes	N/A	N/A	N/A	Yes
Sallum 2014 [18]	Yes	Yes	Yes	Yes	Yes	Yes	Yes	Yes
Schraps 2024 [19]	Yes	Yes	Yes	Yes	Yes	Yes	Yes	Yes
Shersher 2011 [20]	Yes	Yes	Yes	Yes	Yes	Yes	No	Yes
Spitali 2012 [21]	Yes	Yes	Yes	Yes	Yes	Yes	No	Yes
Takasugi 2019 [22]	Yes	Yes	Yes	Yes	Yes	Yes	Yes	Yes
Tranchart 2014 [23]	Yes	Yes	Yes	Yes	Yes	Yes	Yes	Yes
Yancey 2025 [24]	Yes	Yes	Yes	Yes	Yes	Yes	No	Yes

**Note:** Q1 = Were the patient’s demographic characteristics clearly described? Q2 = Was the patient’s history clearly described and presented as a timeline? Q3 = Was the current clinical condition of the patient on presentation clearly described? Q4 = Were diagnostic tests or assessment methods and the results clearly described? Q5 = Was the intervention(s) or treatment procedure(s) clearly described? Q6 = Was the post-intervention clinical condition clearly described? Q7 = Were adverse events or unanticipated events identified and described? Q8 = Does the case report provide takeaway lessons?; N/A = not applicable.

**Table A2:** Clinical goals and achieved doses for organs at risk of the cervical IMRT treatment plan.

Organ/Structure	Priority	Clinical Goal	Achieved Value
Bone_Mandible	P3	Dmax < 7000 cGy	88.56 cGy
Brain	P3	Dmax < 6000 cGy	28.11 cGy
Brainstem	P3	Dmax < 5400 cGy	24.63 cGy
Cavity_Oral	P3	Dmean ≤ 3000 cGy	36.47 cGy
Cochlea_L	P4	Dmax < 6000 cGy	17.10 cGy
Cochlea_R	P4	Dmax < 6000 cGy	15.63 cGy
Eye_L	P4	Dmax < 5000 cGy	12.31 cGy
Eye_R	P4	Dmax < 5000 cGy	12.46 cGy
Glnd_Thyroid	R	V6000 cGy < 100.0%	0.00%
Glnd_Thyroid	R	V4500 cGy < 100.0%	6.56%
Larynx	P4	Dmean < 5000 cGy	2284.68 cGy
Lungs	R	V2000 cGy < 37.0%	1.10%
Lungs	R	Dmean < 2000 cGy	181.62 cGy
Parotid_L	P4	Dmean < 2500 cGy	31.85 cGy
Parotid_L	P4	V3000 cGy < 50.0%	0.00%
Parotid_R	P4	Dmean < 2500 cGy	28.01 cGy
Parotid_R	P4	V3000 cGy < 50.0%	0.00%
SpinalCanal	P3	Dmax < 4500 cGy	2093.99 cGy

**Note:** Dmax = maximum dose; Dmean = mean dose; Vx (e.g., V2000, V3000, V4500, V6000) = percentage of the organ volume receiving at least x cGy; cGy = centigray; L = left; R = right; P3 and P4 indicate treatment-planning priority levels as displayed in the planning system; R indicates a reporting/reference goal.
